# A Comparative Study of Placebo Versus Opioid-Free Analgesic Mixture for Mastectomies Performed Under General Anesthesia Along With Erector Spinae Plane Block

**DOI:** 10.7759/cureus.34457

**Published:** 2023-01-31

**Authors:** Monisha B, Sujatha Munireddy Papireddy, Sreeramulu P N, Sumanth Tarigonda

**Affiliations:** 1 Anesthesiology, Sri Devaraj Urs Medical College, Tamaka, IND; 2 Anesthesia, Sri Devaraj Urs Medical College, Tamaka, IND; 3 Surgery, R L Jalappa Hospital and Research Centre, Tamaka, IND

**Keywords:** opioid-free anesthesia, magnesium sulfate, dexmedetomidine, modified radical mastectomy, ketamine, erector spinae plane block

## Abstract

Background and objectives

Breast cancer is the most frequent cancer among women, globally. Postoperative pain after mastectomy not only causes slow recovery and prolonged hospital stay but can also increase the risk of chronic pain. For patients undergoing breast surgery, effective perioperative pain management is required. Various approaches have been introduced to overcome this, such as opioids, non-opioid analgesics, and regional blocks. The erector spinae plane block is a new regional anesthesia technique used in breast surgery to provide adequate intraoperative and postoperative analgesia. Opioid-free anesthesia is a multimodal analgesia technique that does not use opioids and thus prevents opioid tolerance after surgery. This study aims to investigate whether administering an opioid-free analgesic mixture lowers the pain score and the need for analgesics during and after surgery.

Material and methods

In this randomized prospective comparative clinical study, 66 patients of the American Society of Anesthesiologists (ASA) psychological status (PS) class 1 and 2, aged 18 to 80, were included. Group M received erector spinae plane block + general anesthesia + opioid-free analgesic mixture (1 mcg/cc dexmedetomidine + 1 mg/cc ketamine + 100 mg/cc magnesium sulfate prepared in a 20 ml syringe). Group N received erector spinae plane block + general anesthesia + 20ml of normal saline infusion. The primary outcome was to assess pain scores in the perioperative period. The secondary outcomes were to compare the time for the first rescue analgesia requirement perioperatively, intraoperative hemodynamic profile, and postoperative patient satisfaction. A p<0.05 was considered to be statistically significant.

Results

All patients were females undergoing modified radical mastectomy or breast conservative surgery + axillary sampling + latissimus dorsi flap reconstruction. The visual analog scale (VAS) scores were less than or equal to 3 in zero, first, and second hours postoperatively in both groups. The pain was moderate i.e., less than 4 in almost all time intervals in both groups. Group M had a better intraoperative hemodynamic profile, including mean arterial pressure and heart rate when compared to group N. In group M, the time of request for rescue analgesia was 726.67±390.99 minutes, while it was 468±278.79 minutes in group N. The total analgesic requirement was less in group M than in group N, but this was not statistically significant.

Conclusion

Multimodal analgesia with erector spinae plane block and opioid-free analgesic mixture provides effective perioperative analgesia and a better intraoperative hemodynamic profile in patients undergoing breast cancer surgery under general anesthesia.

## Introduction

Breast cancer is the most common type of cancer in women, worldwide. There were about 2.3 million new cases in 2020 accounting for 11.7% of all cancers, overtaking lung cancer as the leading type of cancer globally [1]. With the high incidence of breast carcinoma, modified radical mastectomy has become one of the most common surgeries [2].

To overcome postoperative pain, multimodal analgesic techniques have been introduced such as opioids, non-opioid analgesics, and regional anesthesia techniques such as thoracic paravertebral block, thoracic epidural block, pectoral nerve (PECS) I and II block, erector spinae plane block (ESPB), serratus anterior plane block, transversus thoracic plane block, intercostal and interpleural nerve blocks. Opioids cause side effects such as opioid tolerance and dependence, postoperative headache, respiratory depression, nausea, pruritis, and urinary retention, and contribute to ileus. Other methods include wound infiltration and local anesthetic installation through surgical drain [3,4].

Mulier investigated opioid-free anesthesia (OFA) in 2009 and the OFA mixture protocol was reported in 2017 [5]. The OFA is an intravenous mixture that includes a combination of one or more drugs such as lidocaine, ketamine, magnesium sulfate, alpha 2 agonists such as dexmedetomidine and clonidine, and non-steroidal anti-inflammatory drugs (NSAIDs). The OFA alone or in combination with regional blocks avoids the side effects of opioids while causing fewer postoperative complications and ensuring speedy recovery, even in oncological surgeries performed under general anesthesia [6].

The ESPB is a novel regional anesthetic technique that has reduced postoperative opioid consumption and improved satisfactory analgesia following mastectomies [7]. The ESPB is simple to perform because of the easy identification of anatomical landmarks on ultrasound. It is relatively safe due to the absence of vital structures in the close vicinity of the block.

The primary objective of this study is to determine whether the administration of an OFA mixture reduces pain scores in the perioperative period. The secondary objectives are to compare the requirement of rescue analgesics in the perioperative period, intraoperative hemodynamic profiles, and postoperative patient satisfaction.

## Materials and methods

We conducted a randomized prospective comparative clinical study in a tertiary care hospital in Kolar, Karnataka, India, from January 2021 to May 2022 after obtaining ethical approval from the institutional ethics committee (approval no. SDUMC/KLR/IEC/606/2020-21).

Sample size

**Figure 1:**
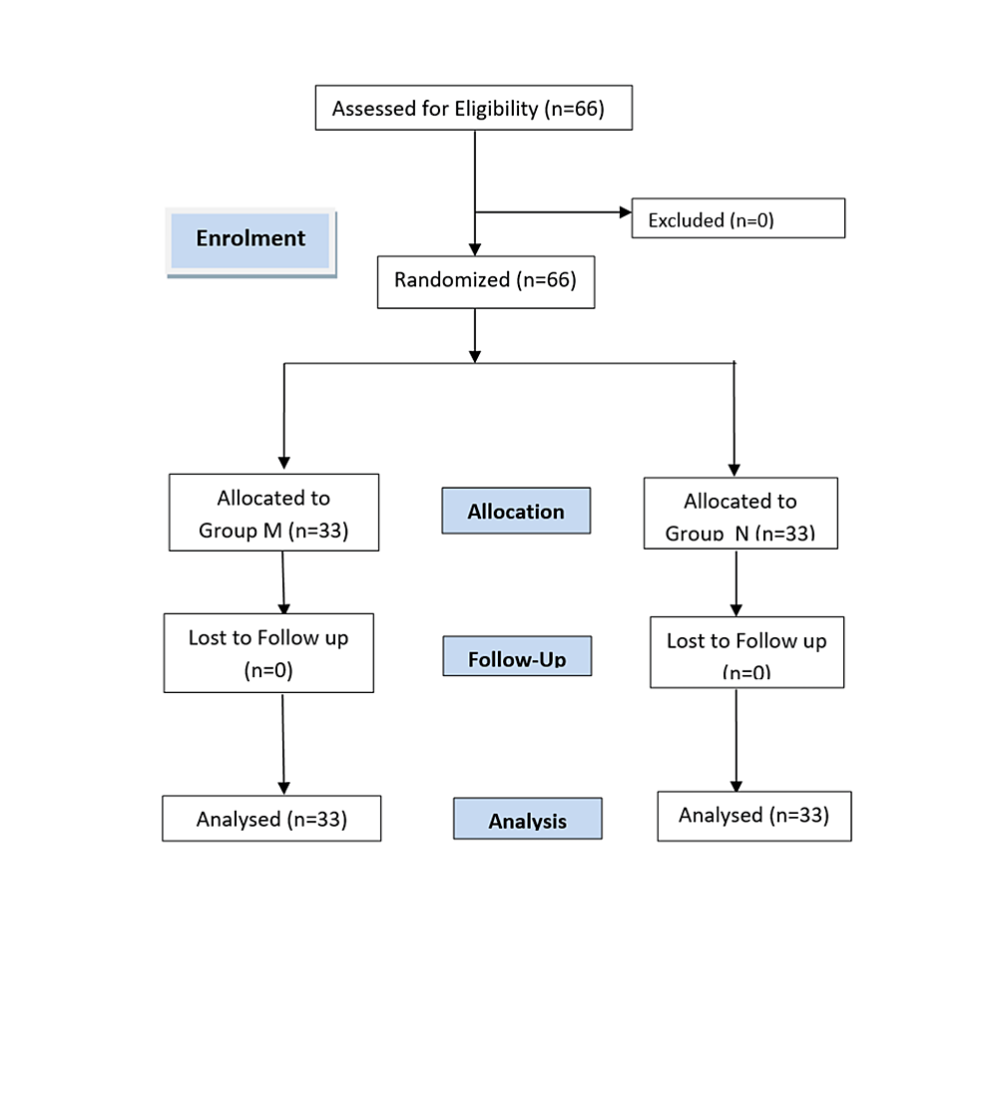
Consolidated Standards of Reporting Trials (CONSORT) flow diagram Group M: Erector spinae plane block + general anesthesia + OFA mixture Group N: Erector spinae plane block + general anesthesia + normal saline

Based on the findings of Singh et al., to detect the difference of 15% reduction in analgesic requirement in a 24-hour postoperative period with ἀ - error of 1%, power of 80% [8], the total sample size of 66 was calculated, with 33 patients in each group (Figure 1).

Female patients aged 18 to 80 years with American Society of Anesthesiologists (ASA) physical status (PS) I and II scheduled for modified radical mastectomy or breast conservative surgery + axillary sampling + latissimus dorsi flap were included in the study. Patients with respiratory and cardiac problems, hepatic and renal problems, coagulopathies, local infection at the injection site, allergy to any of the study drugs used (after the test dose), spine deformities, pregnancy or breastfeeding, severe obesity (BMI>35 kg/m2), and psychiatric illness were excluded from the study. Study participants that satisfied the inclusion and exclusion criteria were randomized, using a computer-generated random number program.

Statistical analysis

The collected data were coded and entered into an excel database. All quantitative measures were presented as (mean+/-standard deviation (SD)), confidence interval, and qualitative measures like gender, ASA physical status, etc., by proportions and confidence interval (CI). Independent sample t-test, chi-square test, Fisher’s exact test, and Mann-Whitney U test were considered appropriate to interpret the results. A p-value <0.05 was considered statistically significant.

The pre-anesthetic evaluation was done for all patients on the day before surgery. Informed consent was obtained from the patient. All patients were given nothing by mouth for solids for eight hours and clear fluids for two hours. All routine investigations were checked. On the day of surgery, an 18G IV cannula was secured on the non-operating hand and IV fluids started. After shifting the patient to the operation theatre, monitors were attached, and basal vitals were documented. Saturation of peripheral oxygen, 5 lead electrocardiogram, heart rate, and non-invasive blood pressure was monitored throughout the surgery. Through computerized randomized sampling, the patients were allotted to one of the study groups (group M or group N).

Group M patients received an OFA mixture (1 mcg/cc of dexmedetomidine + 1 mg/cc of ketamine + 100 mg/cc of magnesium sulfate prepared in a 20 ml syringe) as an intravenous infusion started immediately after intubation @ 1 ml/10 Kg/hr till 30 minutes before extubation.

Group N patients received 20 ml of normal saline loaded in a 20 ml syringe as an intravenous infusion started @ 1 ml/10 Kg/hr till 30 minutes before extubation.

The principal investigator and the patient were blinded to the group to which the patient belongs. Allocation of the group and drug preparation was done by a resident doctor not involved in intraoperative and postoperative monitoring of the patients.

The ESPB was performed at the T4 level in patients of both groups under strict aseptic precautions on the operative side by placing the patient in a sitting position. A linear probe of 5 Hz to 10 Hz was placed in the paramedian longitudinal plane over T4 transverse process. A 23 gauge Quincke needle was used to perform the ultrasound-guided block, using the in-plane approach from the cephalad to the caudal direction. Hydro dissection was done with 2 ml of saline solution to confirm that the needle was in the correct location. Around 25 ml of 0.25% bupivacaine combined with 8 mg dexamethasone was deposited posterior to the tip of T4 transverse processes under the erector spinae muscle [2].

Patients were administered 100% oxygen for three minutes and premedicated with IV glycopyrrolate 0.2 mg. The induction was done with 2 mg/kg to 2.5 mg/kg of propofol following which the ability to ventilate was confirmed and 0.08 mg/kg to 0.1 mg /kg of IV vecuronium was given for muscle relaxation. Patients were ventilated for three minutes with isoflurane 1%, following which intubation was performed with an appropriate-size endotracheal tube. Following confirmation of endotracheal tube placement, anesthesia was maintained with oxygen (O2), nitrous oxide (N2O), isoflurane, and boluses of vecuronium in both groups.

All patients were monitored intraoperatively for hemodynamic changes such as heart rate and mean arterial pressure. Baseline parameters were documented every five minutes till 15 minutes. After 15 minutes, hemodynamic changes were documented every 15 minutes till the end of surgery. Any change in mean arterial pressure and heart rate above 20% of the baseline was documented and rescue analgesic was given with IV tramadol 1 mg/kg or IV diclofenac 75 mg. The IV fentanyl (1 mcg/kg to 2 mcg/kg) was supplemented if the patient did not respond to the above analgesics. All patients were kept under observation in the post-anesthesia care unit (PACU) until an Aldrete score of ≥9/10 before shifting to the ward.

Postoperatively, the patients were assessed for pain in the surgical site, at rest, and on movement using the visual analog scale (VAS) score. The patients were assessed at the zero, first, second, fourth, sixth, 12th, and 24th hour of the postoperative period. A VAS score of more than 3 was considered as the presence of pain and rescue analgesic was given depending on the severity of pain, with IV tramadol 1 mg/kg with or without IV diclofenac 75 mg according to the WHO analgesic ladder. The patients were assessed for overall satisfaction scores using the Likert scale. Postoperative side effects such as nausea and vomiting were recorded.

## Results

This was a prospective randomized comparative clinical study conducted on 66 patients and data collected. All 66 participants were included and divided into 33 participants per group. The age group ranged from 30 to 80 years. The demographic parameters of age and BMI were comparable between the two groups (Table 1). All patients were females posted for modified radical mastectomy or breast-conserving surgery + axillary sampling + latissimus dorsi flap reconstruction.

**Table 1:** Comparison of baseline demographic variables SD: Standard deviation

Variable	Group M (n=33)		Group N (n=33)		p-value
	Mean ± SD		Mean ± SD		
Age (in years)	52.54 ± 12.92		50.96 ± 10.82		0.593
BMI (Kg/m^2^)	22.95 ± 4.11		24.01 ± 3.35		0.257
Surgery	Group M (n=33)		Group N (n=33)		p-value
	Frequency	%	Frequency	%	
Modified Radical Mastectomy	18	54.5%	19	57.5%	0.8
Breast Conservative Surgery + Axillary Sampling + Latissimus Dorsi Flap Reconstruction	15	45.5%	14	42.5%	

In group M, 54.5% of patients underwent a modified radical mastectomy, and 45.5% underwent breast conservative surgery + axillary sampling + latissimus dorsi flap. In group N, 57.5% of patients underwent a modified radical mastectomy, while 42.5% underwent breast conservative surgery + axillary sampling + latissimus dorsi flap reconstruction as shown above in Table 1.

As per the primary outcomes, the VAS scores were significantly lower (p<0.05) in the zero, first, and second hour in group M, compared to group N postoperatively both at rest and on movement (part A and B of Table 2). As shown in the table below, patients had a VAS score of less than three for the first 2 hours, both at rest and on movement. VAS scores at rest were less than three at all the time intervals except at the 6th hour in group N and the 24th hour in group M. Even at this interval, the pain was moderate, with a VAS score of more than 3 but less than 4, which was not clinically significant. The VAS scores on movement were above 3 in both the groups at the fourth, sixth, 12th and 24th hour postoperatively.

**Table 2:** (A) VAS at rest, (B) VAS at abduction, (C) time for first rescue analgesia,  and (D) total analgesic requirement in both groups VAS: Visual analog scale, SD: Standard deviation *Significant, **Highly significant

(A) VAS Score at Rest (in hours)	Group M	Group N	Mean Difference	p-value
	Mean ± SD	Mean± SD		
0	1.33±1.53	2.15±1.18	-0.818	0.018*
1	1.39±1.50	2.42±1.00	-1.030	0.002*
2	1.58±1.28	2.79±1.11	-1.212	<0.001**
4	2.48±1.44	2.94±1.12	-0.455	0.156
6	2.82±1.36	3.15±0.97	-0.333	0.256
12	2.94±1.00	2.76±0.97	0.182	0.456
24	3.09±1.16	2.97±1.05	0.121	0.656
Values are mean and SD; p-value by Mann Whitney U test <0.05 is statistically significant				
(B) VAS Score at Abduction (in hours)	Group M	Group N	Mean Difference	p-value
	Mean ± SD	Mean ± SD		
0	2.00±1.75	2.82±1.33	-2.136	0.036*
1	2.03±1.70	3.27±1.01	-3.604	0.001*
2	2.27±1.26	3.52±1.03	-4.385	<0.001**
4	3.30±1.61	3.79±1.14	-1.412	0.163
6	3.58±1.56	3.88±1.14	-0.901	0.371
12	3.76±1.17	3.73±1.07	0.110	0.913
24	3.85±1.35	3.73±1.15	0.392	0.696
Values are mean and SD; p-value by Man Whitney U test <0.05 is statistically significant				
(C) Time for First Rescue Analgesia (in minutes)	Group M	Group N	Mean Difference	p-value
	Mean ± SD	Mean ± SD		
	726.67±390.99	468.18±278.79	3.092	0.003*
(D) Total Analgesic Requirement (in mg)	Group M	Group N	Mean Difference	p-value
	Mean ± SD	Mean ± SD		
Injection diclofenac	93.00±32.69	116.96±52.72	-23.96	0.055
Injection tramadol	61.90±26.94	69.17±26.00	-7.262	0.338
Values are mean and SD; p-value by Man Whitney U test <0.05 is statistically significant				

The time for the first rescue analgesia request was significantly (p=0.003*) more in group M, with a mean value of 726.67±390.99 minutes, compared to 468±278.79 minutes in group N, as shown above in part C of Table 2. Out of this, 61 patients required rescue analgesia in the first 24 hours. One patient from group N and four from group M did not seek analgesia for more than 24 hours. In group N, the mean amount of diclofenac requirement was 116.96 mg, whereas, in group M, the mean requirement was 93.00 mg with a p-value of 0.055, which is statistically not significant. In group M, the mean amount of tramadol requirement was 61.90 mg, whereas, in group N, it was 69.17 mg with a p-value of 0.338 which is statistically not significant, as shown in part D of Table 2. In group M, 2.48 doses of cumulative analgesic were required, and in group N 2.93 doses of cumulative analgesia were required.

Heart rate did not show a statistical difference between the two groups till 45 minutes after giving the block, but there was a significant statistical difference (p<0.05) from 60 minutes to 225 minutes. But the heart rate in both the groups from 60 minutes was within the normal physiological range; so, it was not significant clinically as shown in Table 3.

**Table 3:** Comparision of mean heart rates between the groups BPM: Beats per minute, SD: Standard deviation * Significant, ** Highly significant

Time in Minutes	Heart Rate in BPM		Mean Difference	T-value	p-Value
	Group M	Group N			
	Mean ± SD	Mean ±SD			
0	82.52±13.60	86.94 ±8.14	-4.424	-1.604	0.114
5	81.91±12.89	87.18 ±11.84	-5.273	-1.731	0.088
10	84.42±11.87	86.85 ±12.03	-2.424	-0.824	0.413
15	82.79±12.10	86.52 ±11.17	-3.727	-1.268	0.209
30	82.73±10.55	85.09 ±11.17	-2.364	-0.859	0.394
45	80.15±11.9	85.91 ±12.37	-5.758	-1.983	0.052
60	80.55±11.15	86.97 ±9.80	-6.424	-2.486	0.016*
75	77.94±10.32	83.24 ±11.11	-5.303	-2.008	0.049*
90	76.30±9.69	81.64 ±11.43	-5.333	-2.044	0.045*
105	75.06±9.78	82.42 ±11.23	-7.364	-2.841	0.006*
120	75.36±8.36	81.24 ±10.10	-5.879	-2.576	0.012*
135	73.67±9.84	80.53 ±11.12	-6.865	-2.638	0.010*
150	73.09±9.48	80.90 ±11.55	-7.809	-2.936	0.005*
165	71.48±8.66	82.61 ±10.73	-11.123	-4.400	<0.001**
180	71.21±8.16	81.64 ±9.71	-10.436	-4.399	<0.001**
195	69.21±8.37	81.36 ±10.72	-12.155	-4.306	<0.001**
210	69.25±7.60	79.41 ±11.29	-10.162	-3.254	0.003*
225	70.00±8.91	77.54 ±10.24	-7.538	-2.184	0.037*
240	68.42±9.87	77.86 ±14.45	-9.440	-1.698	0.108
255	69.56±9.49	66.00 ±9.17	3.556	0.566	0.584
270	69.17±9.02	68.00	1.167	0.174	0.868
285	72.40±8.29	67.00	5.400	0.594	0.584
300	74.50±9.19	68.00	6.500	0.577	0.667
315	77.00±14.14	.			
330	76.00	.			
345	78.00	.			

Mean arterial pressure showed a statistical difference (p<0.05) from 0 minutes to 255 minutes in both groups. Patients in group M showed a lower mean arterial pressure compared to group N up to 255 minutes, suggesting that ESPB along with OFA mixture, provides better blood pressure intraoperatively. Mean arterial pressure in group N was also maintained within the normal range as shown in Table 4.

**Table 4:** Comparison of mean arterial pressure between the groups MAP: Mean arterial pressure, SD: Standard deviation *Significant, **Highly significant

Time in Minutes	MAP in mmHg		Mean Difference	T-value	p-value
	Group M	Group N			
	Mean ± SD	Mean ± SD			
0	86.45±11.82	94.48±10.74	-8.030	-2.888	0.005*
5	86.18±11.38	96.33±10.86	-10.152	-3.707	<0.001**
10	83.33±10.01	96.12±9.43	-12.788	-5.341	<0.001**
15	83.45±8.81	97.39±12.92	-13.939	-5.120	<0.001**
30	82.82±9.97	93.48±9.87	-10.667	-4.367	<0.001**
45	84.61±11.37	94.79±7.36	-10.182	-4.319	<0.001**
60	85.21±11.87	94.09±10.25	-8.879	-3.252	0.002*
75	85.15±10.26	94.67±8.52	-9.515	-4.100	<0.001**
90	85.85±10.95	93.55±9.60	-7.697	-3.037	0.003*
105	83.61±10.30	92.12±11.66	-8.515	-3.145	0.003*
120	83.64±11.06	94.18±9.41	-10.545	-4.172	<0.001**
135	82.82±10.61	94.06±9.21	-11.244	-4.557	<0.001**
150	82.38±9.97	95.52±9.42	-13.141	-5.373	<0.001**
165	83.13±8.44	95.43±10.71	-12.300	-4.924	<0.001**
180	82.00±9.34	93.82±11.60	-11.821	-4.249	<0.001**
195	82.25±10.33	92.14±10.50	-9.886	-3.217	0.002*
210	81.65±8.90	91.24±5.66	-9.585	-3.827	0.001*
225	83.78±7.80	91.46±8.19	-7.684	-2.651	0.013*
240	84.75±11.03	98.86±8.90	-14.107	-2.872	0.011*
255	85.50±9.90	101.00±10.00	-15.500	-2.308	0.046*
270	84.33±11.04	101.50±4.95	-17.167	-2.046	0.087
285	80.80±10.69	103.00	-22.200	-1.896	0.131
300	80.50±4.95	105.00	-24.500	-4.041	0.154
315	85.00±21.21				
330	65.00				
345	70.00				

In group M, 54.5% of patients were very much satisfied, and in group N 45.5% of patients were somewhat satisfied. Around 33.3% of patients from group M were somewhat satisfied and 36.4% of patients were not able to decide in group N. This shows patients in group M had better satisfaction scores than group N which was statistically significant as shown in Table 5. None of the patients had side effects like nausea and vomiting in the postoperative period till 24 hours.

**Table 5:** Comparison of patient satisfaction scores between the groups *Significant.

Patient Satisfaction Score	Group M		Group N		p-value
	Frequency	%	Frequency	%	
5 (very much satisfied)	18	54.5%	5	15.2%	0.004*
4 (somewhat satisfied)	11	33.3%	15	45.5%	
3 (undecided)	3	9.1%	12	36.4%	
2 (not really satisfied)	1	3.0%	1	3.0%	
1 (not at all satisfied)	0	0.0%	0	0.0%	

## Discussion

Patients undergoing breast cancer surgery experience severe pain, which affects their quality of life. To reduce postoperative pain, various techniques can be used such as intravenous analgesics, opioids, and regional anesthesia. With the use of ultrasound, regional anesthetic techniques have become more popular because of better visualization of anatomical structures, which helps in the administration of local anesthetics under the vision and reduces the risk of block failure. Much research has been done to improve analgesia in patients undergoing modified radical mastectomy [9].

As opioid-based general anesthesia alone cannot provide postoperative analgesia, regional anesthesia has gained popularity in recent years as it reduces postoperative pain and improves patient comfort. In our study, we used Mulier’s mixture (ketamine, magnesium sulfate, and dexmedetomidine) as an adjunct to regional anesthesia to provide OFA. We preferred ESPB over other regional anesthetic techniques because of its ease of administration and fewer resultant complications. The OFA was preferred because opioids are related to postoperative adverse effects.

Postoperative pain scores in patients who received an OFA mixture were significantly less compared to the control group, both at rest and on movement for the first two hours after surgery. Between three to 24 hours post-surgery, there was no statistically significant difference between the groups (parts A and B of Table 2). Kim et al. assessed the efficacy of ESPB along with opioid-sparing analgesia in patients undergoing breast-conservative surgery. Around 20 ml of 0.375% ropivacaine was given for block at the T4 level. Median pain scores were noted at the second, fourth, 12th, 24th, and 48th hour, and the pain was assessed at the breast and axilla region. Median pain scores were less in the ESPB group than in the control group that did not receive block at all the time intervals, but the pain at the axilla was more at all intervals. They also opined that ESPB had a significantly greater effect in reducing breast pain at the 12th, 24th, and 48th hour postoperatively, but not the pain at the axillary region [10].

In a study done by He et al., lower VAS scores were reported up to 48 hours both at rest and on movement in patients who received ESPB along with general anesthesia as compared to patients who received general anesthesia alone. The VAS scores were less than 4 at all time intervals in the ESPB group whereas VAS scores were above 3 at all the time intervals in the control group. They stated that ESPB along with general anesthesia was effective in relieving pain in the axillary region as well as the chest wall postoperatively compared to general anesthesia alone [11].

Majumdar et al. found the mean time for the first rescue analgesic requirement to be 871.30 ± 58.51 minutes in the ESPB group in which patients received 20 ml 0f 0.2% ropivacaine along with 0.5 mcg/kg of dexmedetomidine. In the PECS group, it was 460.00± 507.40 minutes, in which patients received 30 ml of 0.2% ropivacaine along with 0.5 mcg/kg of dexmedetomidine. Compared to both groups, ESPB provided analgesia for a longer duration than the PECS block. The mean time for the first rescue analgesia requirement was 468.2 ± 80 minutes in the study of Elfadel et al., in which one group received ESPB with 0.25% bupivacaine along with general anesthesia and the other group received general anesthesia with intravenous analgesia. In our study, group M took 726.67±390.99 minutes and group N 468±278.79 minutes, which shows that the addition of the OFA mixture as an adjunct to ESPB had provided prolonged postoperative analgesia compared to ESPB alone (part C of Table 2) [12,13].

The total analgesic requirement in our study in the first 24 hours after surgery was less in those who received OFA mixture along with ESPB (part D of Table 2). In a study by Altiparmak et al., (ESPB with 20 ml of injection bupivacaine with different concentrations i.e., 0.25% and 0.375%) where the rescue analgesic given was injection tramadol through patient-controlled analgesia (PCA) pump, patients who received 0.375% required 149.52 ± 25.39 mg and those who received 0.25% required 199.52 ± 32.78 mg of injection tramadol and none of the patients required opioids. In our study, the addition of dexamethasone to 25 ml of 0.25% bupivacaine reduced the requirement of analgesics, and the intravenous infusion of OFA mixture along with ESPB further reduced the requirement of analgesics in the perioperative period [14].

Singh et al. compared the efficacy of ESPB by giving 20 ml of 0.5% bupivacaine where out of 40 patients only three required an injection of morphine 24 hours postoperatively, compared to patients who underwent surgery under general anesthesia [8]. Comparatively, in our study, we preferred the injection of tramadol and injection diclofenac over opioids for postoperative analgesia to avoid opioid-related side effects. None of the patients had side effects such as nausea, vomiting, and bradycardia [8].

Overall, there was not much difference in the mean arterial pressures and heart rate clinically in the intraoperative period, though there was a significant difference statistically. In both groups, the mean arterial pressure and heart rate were within the normal physiological range. Patients in group M who received the OFA mixture had stable intraoperative hemodynamic profiles compared to group N (Tables 3 and 4 ). In a study done by Elewa et al., a comparison was made between the analgesic efficacy of ESPB, paravertebral block, and general anesthesia alone. This study stated that both techniques provided superior analgesia and a better hemodynamic profile when compared to general anesthesia alone [15].

Most of the patients in our study had satisfaction scores of 4 and 5 in group M, whereas in group N it was 3 and 4 which shows patients' satisfaction was more in group M compared to group N (Table 5).

In our study, in group M, an OFA mixture was used, which decreased the total analgesic requirement intraoperatively and hemodynamics were stable, compared to group N. In a case report by Sarma et al., however, it was indicated that five patients received ESPB with 2% ropivacaine along with OFA which included injection dexmedetomidine 0.5 mcg/kg initiated 10 minutes before induction and 40 mg/kg magnesium sulfate infusion over 10 minutes after induction. If hemodynamics were unstable, they increased the injection of dexmedetomidine infusion and gave an injection of ketamine 10 mg to reduce the response. They concluded that OFA can be considered an effective substitute for opioid-based anesthesia [16].

Strengths and limitations

Our study is the first randomized control study to evaluate the combination of OFA mixture along with ESPB for mastectomies. The sample size was relatively adequate to draw a conclusion. However, there are a few limitations. The block was performed when patients were awake and general anesthesia was given as soon as the block was performed. In our study, we have not assessed the effect of the block and detected block failures.

Another limitation was the Covid pandemic, due to which we could not get an adequate number of modified radical mastectomy cases. We have hence included breast conservative surgery + axillary sampling + latissimus dorsi flap reconstruction cases. With the volume of drugs used in our study, adequate analgesia would not have been provided in surgeries involving latissimus dorsi flap reconstruction.

## Conclusions

Infusion of OFA mixture (combination of ketamine, magnesium sulfate, and dexmedetomidine) as an adjunct to ESPB reduces the postoperative pain scores, prolongs the duration of postoperative analgesia as well as reduces the requirement of rescue analgesics in the postoperative period.

Intraoperative hemodynamics and patient satisfaction scores were significantly better in those patients who received an OFA mixture without any side effects or complications. To conclude, intravenous administration of OFA mixture improves the quality of perioperative analgesia in patients undergoing mastectomies under ESPB-based general anesthesia.
